# Self-aggregating TIAF1 in lung cancer progression

**DOI:** 10.1186/2213-0802-1-5

**Published:** 2013-02-28

**Authors:** Qunying Hong, Li-Jin Hsu, Pei-Yi Chou, Ying-Tsen Chou, Chen-Yu Lu, Yu-An Chen, Nan-Shan Chang

**Affiliations:** 1 Department of Pulmonary Medicine, Zhongshan Hospital, Fudan University, Shanghai, Peoples’ Republic China; 2 Department of Medical Laboratory Science and Biotechnology, National Cheng Kung University College of Medicine, Tainan, Taiwan; 3 Institute of Molecular Medicine, National Cheng Kung University College of Medicine, Tainan, Taiwan; 4 Institute of Basic Medical Science, National Cheng Kung University College of Medicine, Tainan, Taiwan; 5 Advanced Optoelectronic Technology Center, National Cheng Kung University, Tainan, Taiwan; 6 Department of Neurochemistry, New York State Institute for Basic Research in Developmental Disabilities, Staten Island, New York, NY USA; 7 Department of Neuroscience and Physiology, SUNY Upstate Medical University, Syracuse, NY USA

**Keywords:** TGF-β, TIAF1, Protein aggregation, Lung cancer

## Abstract

Recent studies have demonstrated that transforming growth factor beta (TGF-β1)-induced antiapoptotic factor (TIAF1) is able to form aggregates in the hippocampi of middle-aged normal individuals. The aggregating TIAF1 induces generation of amyloid beta (Aβ) for causing neurodegeneration. Intriguingly, TIAF1 aggregates are shown, together with Smad4 and Aβ, in the cancer stroma and peritumor capsules of many solid tumors. During lung cancer progression, for example, TIAF1 and amyloid fibrils are significantly upregulated in the cancer stroma. Aggregates of TIAF1 and Aβ are shown on the interface between metastatic lung cancer cells and the brain tissues. Conceivably, these peritumor materials are needed for cancer cells to survive. *In vitro* experiments revealed that TIAF1 is a crucial component for tumor suppressors p53 and WWOX-mediated tumor suppression and apoptosis. While metastatic lung cancer cells are frequently devoid of WWOX and p53, we provide new perspectives regarding the role of TIAF1 in the pathogenesis of lung cancer development, and propose a therapeutic approach for targeting TIAF1.

**Electronic supplementary material:**

The online version of this article (doi:10.1186/2213-0802-1-5) contains supplementary material, which is available to authorized users.

## Tumor suppressors WWOX and p53

**Figure 1:**
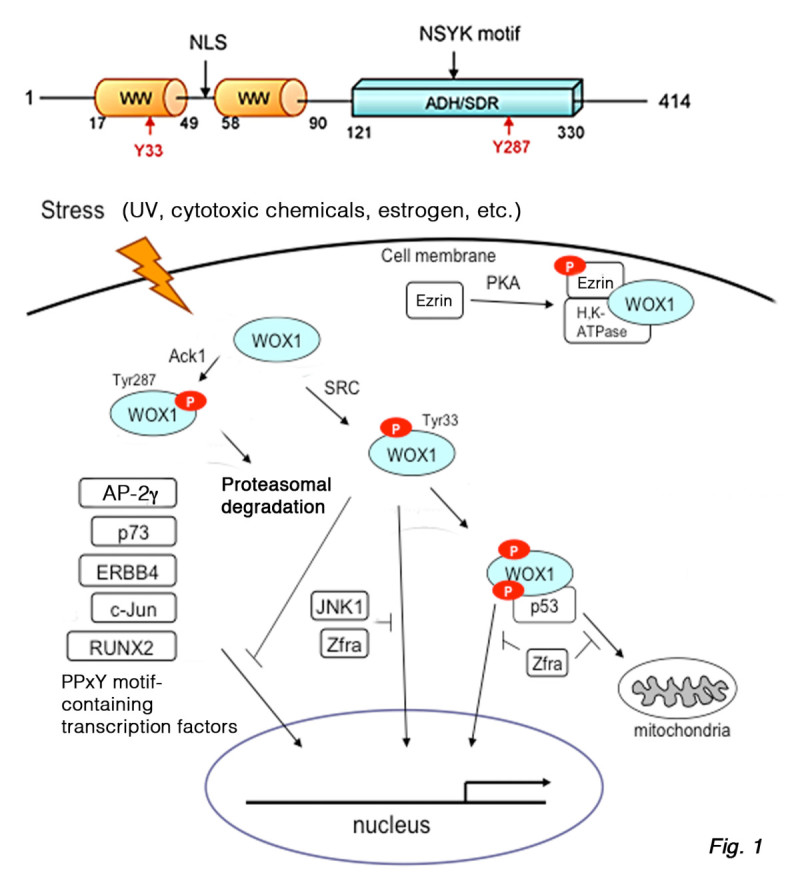
**WWOX signaling.** The full-length WWOX or WOX1 has two *N*-terminal WW domains and a *C*-terminal short-chain alcohol dehydrogenase/reductase (ADH/SDR) domain [1–5]. A nuclear localization signal (NLS) is located between the WW domains. Sex steroid hormones may interact with the NSYK motif in the ADH/SDR domain [4, 5]. Under stress stimuli, tyrosine kinase SRC and probably other kinases induce WWOX activation via Tyr33 phosphorylation. Activated WWOX binds Ser46-phosphorylated p53, and relocates to the mitochondria and nuclei to induce apoptosis. JNK1 and Zfra bind WWOX and counteract its-mediated apoptosis. The first WW domain of WWOX interacts with PPxY motif-containing transcription factors, including AP-2γ, p73, ERBB4, c-Jun and RUNX2. The binding allows transiently overexpressed WWOX to prevent relocation of transcription factors to the nucleus *in vitro*. However, the event does not work *in vivo*[6]. Phosphorylated Ezrin binds and anchors WWOX to the membrane/cytoskeleton area. Activated tyrosine kinase ACK1 phosphorylates WWOX at Tyr287 for polyubiquitination and proteosomal degradation.

Tumor suppressors p53 and WWOX have been shown to play critical roles in apoptosis and control of cancer progression [1–3]. WW domain-containing oxidoreductase, designated as WWOX, FOR, or WOX1, is encoded by human or mouse *WWOX*/*Wwox* gene. The full-length WWOX protein is composed of two *N*-terminal WW domains and a *C*-terminal short-chain alcohol dehydrogenase/reductase (SDR) domain (Figure 1) [3]. WWOX may act as an alternative receptor for sex steroid hormones, since its SDR domain possesses a hormone-binding NSYK motif [4, 5]. The first WW domain interacts with proteins possessing a PPxY-motif(s), including AP-2, p73, ErbB4, Ezrin, SIMPLE, c-Jun, RUNX2 and many others [3]. Transiently overexpressed WWOX prevents the relocation of transcription factors AP-2, p73, ErbB4, c-Jun and RUNX2 to accumulate in the nuclei *in vitro*. However, the observations *in vitro* do not appear to be true *in vivo*. Activated Wwox with Tyr33 phosphorylation, along with transcription factors c-Jun, CREB, JNK1, ATF3 and NF-κB, rapidly undergoes relocation to the nuclei in axotomized neurons in sciatic nerve transection in rats [6]. When WWOX undergoes activation via phosphorylation at Tyr33 (probably by SRC kinase), it binds a broad spectrum of proteins without the PPxY motif [3, 5].

The domain structure of p53 includes a natively unfolded *N*-terminal transactivation domains TAD1 and TAD2, a proline-rich region (PRR), a central DNA-binding domain, and a *C*-terminal tetramerization domain [2, 7]. Activated p53 causes cell cycle arrest, initiates DNA repair, and may lead to apoptosis [7]. p53 transactivates cyclin-dependent kinase inhibitor p21 or microRNA miR34 to induce cell cycle arrest [7]. For apoptosis, p53 transactivates proapoptotic genes such as *BAX, PUMA, SCOTIN*, and *FAS*, and inhibits the antiapoptotic gene *BCL2* [7]. Under stress conditions (e.g. UV irradiation and chemotherapeutic drugs), activated WWOX physically binds Ser46-phosphorylated p53. This binding allows WWOX to stabilize p53, and both proteins act together in causing apoptosis [3, 5] (Figure 1).

## Role of p53 and WWOX in blocking cancer initiation and progression

Cancer initiation and progression is generally considered as a consequence of gene mutation or epigenetic inactivation of tumor suppressors. It is believed that mutations in 2 tumor suppressor genes cause cancer – the so-called “2-hit hypothesis” [8]. Recent development revealed that even partial inactivation of tumor suppressors critically contributes to tumorigenesis [8]. Despite these, it is not surprising to find that many tumor suppressor proteins, e.g. p53, WWOX, Smad4, and others, are significantly upregulated during the early stage of cancer progression [3, 9, 10]. Functional significance of these proteins in blocking cancer progression at the early stage is largely unknown. However, a good possibility is that these proteins are functionally inactivated.

Proapoptotic p53, for example, is functionally inactivated by fortilin, an anti-apoptotic protein [11, 12]. Fortilin physically interacts with the sequence-specific DNA binding domain of p53. Oncogenic monocarboxylic acid transporter 1 (MCT-1) abolishes the p53 function by enhancing its degradation via the ubiquitin/proteasome system [13]. Under stress conditions, NF-κB acts as an oncoprotein to promote cell division and survival and block the proapoptotic function of p53 [14]. Mdm2 and Mdmx abolish the stability of p53 [15]. Mdm2 is an E3 ubiquitin ligase that causes p53 ubiquitination and proteasomal degradation. In contrast, Mdmx does not induce p53 degradation, but inhibits p53 by masking its transcriptional activation domain. When WWOX undergoes Tyr33 phosphorylation, the activated protein binds and stabilizes Ser46-phosphorylated p53 [3, 16]. cJun *N*-terminal kinase (JNK1) binds and functionally counteracts with WWOX in apoptosis [17–20]. When JNK1 is blocked by chemical inhibitors (e.g. SP600125), WWOX-mediated apoptosis is enhanced. Zfra (zinc finger-like protein that regulates apoptosis) participates in the mitochondrial apoptosis and binds WWOX to its *N*-terminal first WW domain and *C*-terminal SDR domain, which results in functional antagonism in causing apoptosis [21, 22]. Together, functional antagonism is well known among proapoptotic and apoptotic proteins. It is still not clear whether there is a direct functional antagonism between defined tumor suppressors.

## Silencing *WWOX* gene promoter by hypermethylation

Promoter hypermethylation is believed to block protein expression, and epigenetic control can touch over a large region of chromosomes in a coordinated manner [23, 24]. Promoters for tumor suppressor genes *WWOX* and *Fhit* are frequently hypermethylated and silenced in metastatic cancers [25, 26]. Alternatively, tumor suppressors are inactivated due to gene mutation, fragmentation, or interchromosomal recombination at the later cancer progression and metastasis stages. Nonetheless, expression of tumor suppressor WWOX is increased during the early stages of cancer initiation and growth, and is then dramatically reduced at the late stages of differentiation in skin cancer both in humans and mice [3, 10]. Translational blockade is shown to be involved in the downregulation of WWOX/Wwox protein in humans and mice [10].

## Lack of WWOX and increased migration in metastatic cancers

In most cases, metastatic cancer cells do not have functional tumor suppressors such as WWOX and p53 [3, 4, 25, 26]. How tumor suppressors affect normal cell migration and cancer cell metastasis is largely unknown. Ectopic WWOX affects ovarian cell attachment and migration on fibronectin-enriched extracellular matrix, and, conversely, loss of WWOX facilitates ovarian cell migration [27]. While epithelial-mesenchymal transition is an essential step toward cancer cell metastasis, it appears that many signal pathways converge on several transcription factors, including zinc finger proteins Snail and Slug, Twist, ZEB 1/2, and Smads, and the event results in the morphological transition and metastasis [28].

Cancer cell metastasis is affected by the surrounding tumor microenvironments. Metastatic lung cancer cells appear to be able to detect alterations in the environmental cues, so that they successfully migrate to target organs and settle properly [29, 30]. Non-small-cell lung carcinomas (NSCLC) frequently migrate to the contra-lateral lung, the brain and to organs such as adrenal glands, liver and bones [29]. Whether lung cancer cells specifically select susceptible sites in the endothelial lining of blood vessels for docking and penetration into organs is largely unknown.

Many types of metastatic cancers utilize the CXCR4/CXCL12 signaling axis to reach the targets [31–33]. High levels of functional CXCR4 receptors are expressed in small-cell lung cancer (SCLC) cells. Binding of chemokine stromal derived factor-1 (SDF-1/CXCL12) to the CXCR4 receptors increases the expression of integrin-mediated adhesion in SCLC to target cells.

## TIAF1-p53-WWOX is an axis of tumor suppression

TIAF1 is a transforming growth beta (TGF-β)-induced 12-kDa protein [34–38]. This protein was originally shown to participate in the regulation of the tumor necrosis factor (TNF) pathway. TIAF1 also controls the signaling of TNF receptor adaptor proteins such as TRADD (TNFR1-associated death domain protein), FADD (FAS-associated death domain-containing protein), and RIP (receptor-interacting serine-threonine kinase) [38]. As a TGF-β-induced protein, TIAF1 acts similarly to that of TGF-β1 [37, 38]. For example, TGF-β1 and transiently overexpressed TIAF1 support the growth of fibroblasts [37]. However, epithelial and monocytic cells, as well as many non-fibroblasts, are sensitive to the growth suppression and apoptosis by TGF-β1 and transiently overexpressed TIAF1. In monocytic U937 cells, for example, ectopic TIAF1 increases the expression of p53 and Cip1/p21 and suppresses ERK phosphorylation, which leads to cell growth inhibition and apoptosis [36, 37].

TIAF1 is essential for cell death caused by tumor suppressors p53 and WWOX and dominant-negative JNK1 [36]. When TIAF1 expression is knocked down by small interfering RNA (siRNA), UV irradiation-mediated p53 activation, via Ser15 phosphorylation and nuclear accumulation, is blocked [36]. Importantly, transiently overexpressed TIAF1, p53 and WWOX work synergistically in suppressing anchorage-independent growth, blocking cell migration, and causing apoptosis *in vitro* [34]. When one of the components is missing, the apoptotic function of the TIAF1-p53-WWOX trio is significantly reduced, suggesting that the complex is an axis of tumor suppression [34].

## p53, WWOX and TIAF1 in the TGF-β signaling and SMAD-responsive promoter activation

**Figure 2:**
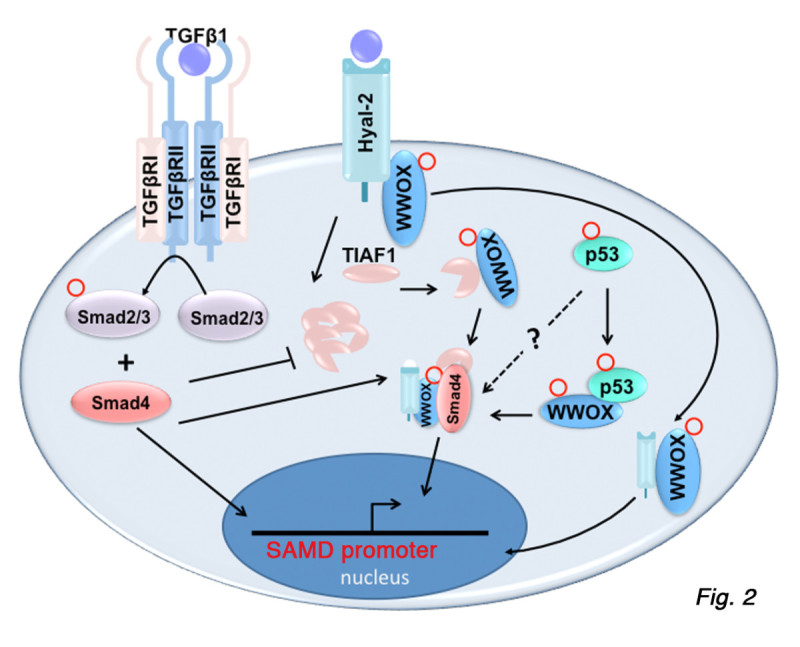
**TGF-** β**1-initiated signal pathways that converge to WWOX, TIAF1, p53, Hyal-2, and Smad4.** TGF-β1 initiates several signal pathways: **i**) In the canonical signaling, extracellular TGF-β1 binds TβRII, followed by recruiting TβRI for subsequent activation and complex formation of Smad2, 3 and 4. The Smad2/3/4 complex mediates gene transcription in the nucleus [39]. **ii**) Alternatively, TGF-β1 utilizes membrane hyaluronidase Hyal-2 as a receptor for signaling the complex formation of Hyal-2 with WWOX and Smad4 to control SMAD-responsive promoter activation [41]. **iii**) Additionally, TGF-β1 induces the complex formation of TIAF1 with Smad4 to regulate the SMAD-responsive promoter activation [35]. **iv**) WWOX binds and prevents p53 degradation and both proteins exert apoptosis synergistically [2, 3]. p53-WWOX-TIAF1 is an axis of tumor suppression [34, 36]. TGF-β1 is likely to converge the TGF-β1-initiated signal pathways to the complex formation of WWOX, TIAF1, p53, Hyal-2, and Smad4. TIAF1 undergoes aggregation during aberrant TGF-β signaling [35]. WWOX: blue; Hyal-2: light blue; TIAF1: pink; p53: light green; Smad4: orange. Red circle: phosphorylation.

p53, WWOX and TIAF1 participate in the pathway of TGF-β/Smad signaling. In the canonical signaling, membrane type II TGF-β receptor (TβRII) is responsible for binding extracellular TGF-β1, β2 or β3, followed by recruiting TβRI and subsequent activation and complex formation of Smad2, 3 and 4. TβRII is a constitutively active serine/threonine kinase. Upon stimulation with TGF-β, TβRII phosphorylates and binds the serine/threonine kinase TβRI. Then, the receptor complex, containing two TβRII and two TβRI, phosphorylates Smad2 and 3 for subsequent recruiting Smad4. The Smad2/3/4 complex translocates to the nucleus to mediate gene transcription [39] (Figure 2). Mutant p53 binds *T* β*RII* promoter to block the expression of TβRII [40]. However, wild type p53 and Smad proteins interact with *AFP* and *Mix2* promoters for either enhancing or suppressing the promoter activity [40]. We reported that TGF-β1 binds membrane hyaluronidase Hyal-2, and the binding leads to complex formation of Hyal-2 with WWOX and Smad4 for regulating the SMAD-responsive promoter activation [41] (Figure 2). Excessive SMAD-regulated promoter activation induces cell death [41]. Most recently, we determined that TGF-β1 signals the binding of TIAF1 with Smad4, and the complex relocates to the nucleus to modulate gene transcription governed by Smad4 [35]. Overall, TGF-β1-initiated signal pathways are likely to converge to the complex formation of WWOX, Hyal-2, TIAF1, p53, and Smad4 for regulating the promoter activation governed by SMAD (Figure 2).

TIAF1 binds Smad4 of the TGF-β/Smad signaling, and that transiently overexpressed TIAF1 blocks SMAD-dependent promoter activation [34]. When TIAF1 is knocked down by siRNA, spontaneous activation of Smad proteins occurs as their accumulation in the nucleus [34]. In parallel, when TIAF1 level is low, spontaneous activation of the promoter governed by the SMAD complex occurs [34]. Most intriguingly, in the absence of endogenous p53, TIAF1 undergoes self-aggregation and simultaneously activates the SMAD-regulated promoter [35]. In parallel with the protein expression of WWOX [10], TIAF1 is upregulated in developing tumors, but may disappear in established metastatic cancer cells [34]. As an axis of tumor suppression, the absence of WWOX-p53-TIAF1 trio favors the growth of metastatic cancer cells.

TIAF1 does not directly interact with p53 [34, 36]. However, TIAF1 is needed for UV irradiation-induced p53 activation (with Ser15 phosphorylation) and nuclear accumulation [36]. Without TIAF1, no p53 activation occurs. WWOX physically binds p53 and stabilizes its function [10]. Data from both yeast two-hybrid and Förster resonance energy transfer (FRET) analyses revealed that WWOX acts as a bridge for the indirect interaction between p53 and TIAF1 (Chang et al., unpublished).

TGF-β1 induces the differentiation of regulatory T cells (Treg), and that TIAF1 expression is significantly increased during cell differentiation [42]. TIAF1 is considered as one of the Treg signature proteins [42]. TIAF1 is also involved in allograft rejection, as its level is significantly increased in activated Th2 helper T lymphocytes in patients with chronic kidney and liver allograft rejection [43]. In addition, TIAF1 is associated with Hirschsprung's disease, a congenital complex disorder of intestinal innervation [44].

## TGF-β, microenvironment and cell-cell contacts affect TIAF1 aggregation

In response to TGF-β1 or -β2, TIAF1 undergoes self-aggregation in the cultured cells [34]. The event can occur independently of the canonical signaling from TβRII/RI/Smads [34]. TIAF1 aggregation occurs probably as a result of an aberrant TGF-β signaling in cancer cells, as well as in the hippocampus [34, 35]. Also, when cells are exposed to environmental stress, TIAF1 undergoes self-aggregation, which exhibits as a ladder-like protein polymerization pattern in SDS-PAGE [34]. The overly aggregated TIAF1 induces degradation of amyloid precursor protein (APP), super-induction of Aβ, and ultimately cell death *in vitro* [34]. Caspase inhibitors block the cell death, suggesting that caspases participate in the TIAF1-induced apoptosis.

Microenvironment and cell-cell contacts affect the TGF-β1 induction of endogenous TIAF1 expression and self-aggregation. Also, TGF-β regulates cancer cell and stroma interactions, and that controls the progression of cancer growth [45]. When lung cancer cells are grown on an extracellular matrix (ECM) derived from another cell type, the lung cells have apparently received the environmental stress that leads to the generation of endogenous TIAF1 and Aβ aggregates [34]. No ubiquitination is involved in the TIAF1 polymerization. Intriguingly, the polymerizing TIAF1 increases the expression of tumor suppressors Smad4 and WWOX [34]. And, WWOX in turn upregulates the TIAF1 expression [34]. Smad4 remains a monomer, whereas WWOX may become polymerized as well. Dominant negative TIAF1 is able to block WWOX expression [34].

Furthermore, when fibroblasts and neuroblastoma cells were co-cultured on the ECM of prostate DU145 cells for at least 48 hr, endogenous TIAF1 expression and aggregation were demonstrated in both cells [34]. No TIAF1 aggregate formation was observed by culturing fibroblasts or neuroblastomas alone on the ECM [34]. Additionally, TIAF1 aggregates are present on the interface between metastatic lung cancer cells and the brain cells *in vivo* [34]. Thus, when two distinct types of cells encounter each other in an unfavorable microenvironment, endogenous TIAF1 aggregation occurs.

## TIAF1 aggregation occurs in tumor progression and metastasis *in vivo*

**Figure 3:**
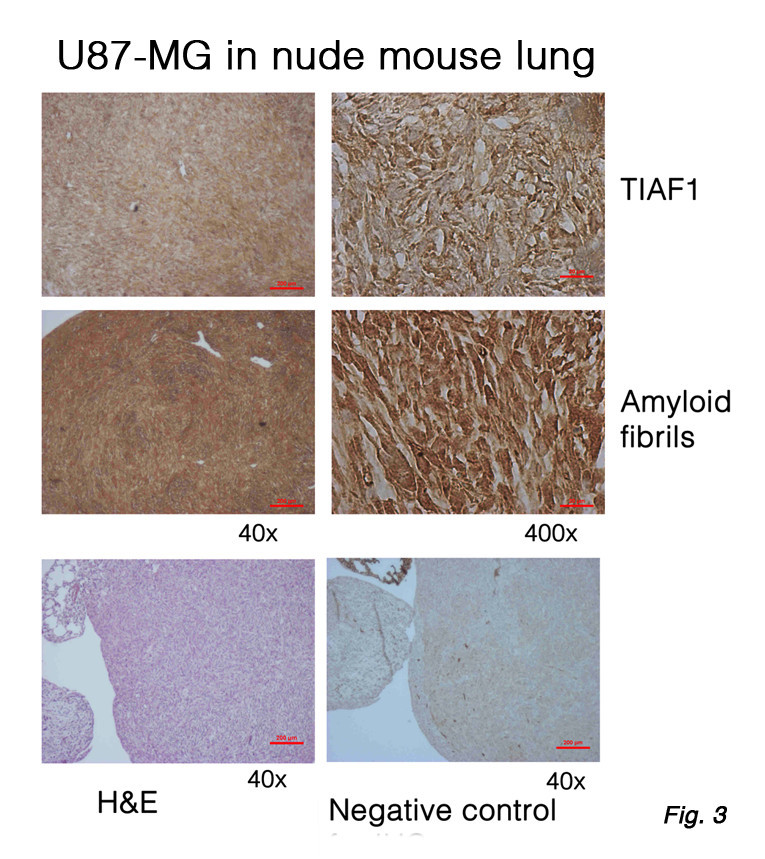
**Expression of TIAF1 and amyloid fibrils in U87-MG glioma cells in the lung.** U87-MG glioma cells were inoculated in both flanks of nude mice. Two months later, the mice were sacrificed. Shown is the U87-MG cell metastatic to the lung. Each solid tumor lesion has increased expression of TIAF1 and amyloid fibrils, compared to normal mice (data not shown). Both proteins appear to localize intracellularly. A homemade TIAF1 antibody and commercial amyloid fibril antibody were used, as described [34, 35].

TIAF1 participates in the initiation of Alzheimer’s disease [35]. We have determined that hippocampal TIAF1 aggregation is shown at ages 40–70, and this event occurs prior to the generation of Aβ plaques in Alzheimer’s disease at 75–90 years old [35]. In parallel with this finding, *in vitro* analysis showed that aggregating TIAF1 causes APP degradation and generation of Aβ [35]. Whether aggregating TIAF1 induces caspase activation for leading to APP degradation is unknown. Upregulation of TIAF1 is also shown during cancer progression and metastasis [34]. When metastatic U87-MG glioma cells, for instance, were subcutaneously inoculated in both flanks of nude mice, the cells were metastatic to the lung. Expression of TIAF1 and amyloid fibrils is shown in the growing solid tumor (Figure 3).

## TIAF1 aggregates in the metastatic lung cancer cells in the brain

It appears that cancer cells could tolerate more microenvironmental stress than normal cells, as cancer cells tend to utilize local or autocrine TGF-β in building up their own niche. Conceivably, upon reaching and selectively attaching to the docking site in the brain, a single metastatic lung cancer cell must face the challenge of survival. The cell needs to divide and proliferate quickly, and build itself own niche by utilizing available sources from the brain tissue or its own TGF-β. The concern is whether the brain tissue recognizes the cancer cell as foreign. The local glial cells or immune cells may come to attack the invading cancer cell. A successfully built solid tumor normally possesses a peritumor capsule. For example, when metastatic small-cell lung cancer cells migrate to the brain, TIAF1 and Aβ aggregates are shown on the border between cancer and brain cells. Or, the protein aggregates are present in the tumor mass [34].

During solid lung tumor formation in the brain, ECM proteins continue to accumulate and they can organize orderly in the matrix or become aggregated in an irregular manner [34]. The ECM aggregates are located inside the tumor mass, or may be present on the borderline between brain cells and the metastatic lung cancer [34]. Quite frequently, TIAFI and Aβ are present in these protein aggregates. Self-aggregation of TIAF1 has been shown to induce phosphorylation of APP, followed by generation of Aβ and amyloid fibrils [35]. The resulting Aβ fails to cause cancer cell death, whereas it is likely to make damage to neurons or initiation of neurodegeneration. Indeed, no apoptotic lung cancer cells have been shown within the peritumor protein aggregates [34], suggesting that these protein aggregates act as a “protective shield” for the progression of lung cancer cells in the brain.

Intriguingly, when metastatic cancer cells have well established their niche, intracellular TIAF1 disappears significantly in the tumor masses [34]. However, TIAF1, Smad4, Aβ, and many other proteins are components of the fibrous aggregates in the peritumor capsules. The extracellular aggregated TIAF1 and Smad4 are probably functionally inactive.

## Concluding remarks

TGF-β1 induces TIAF1 self-aggregation, and the aggregating proteins bind Smad4 and cause Aβ generation. These protein aggregates are likely to induce apoptosis via activation of caspases, as caspase inhibitors can block the cell death [35]. However, cancer cells appear to successfully survive from the stress. The TIAF1-Smad4-Aβ protein aggregates are frequently seen in the peritumor capsules, which are crucial for solid tumor growth and protection. However, the relative concentrations of TIAF1 and Smad4 must be balanced. If cells have a greater amount of TIAF1 than Smad4, they survive upon challenge with TGF-β1. However, when cells express a greater amount of Smad4 than TIAF1, they are highly sensitive to TGF-β1-induced apoptosis [35].

During cancer progression, the expression profiles for WWOX and TIAF1 appear similarly. Both proteins are significantly upregulated during the early phases of benign tumor formation. When tumors acquire their metastatic potential, both proteins are dramatically reduced. WWOX induces the expression of TIAF1, and enhances the transcriptional activation of promoters governed by SMAD and NF-κB [34, 35]. TIAF1 in turn induces the expression of Smad4 and Aβ, depending upon the status of TIAF1 self-aggregation. Intriguingly, under p53-free environment, TIAF1 starts to undergo self-aggregation and the aggregating TIAF1 polymer activates the SMAD-governed promoter [35].

**Figure 4:**
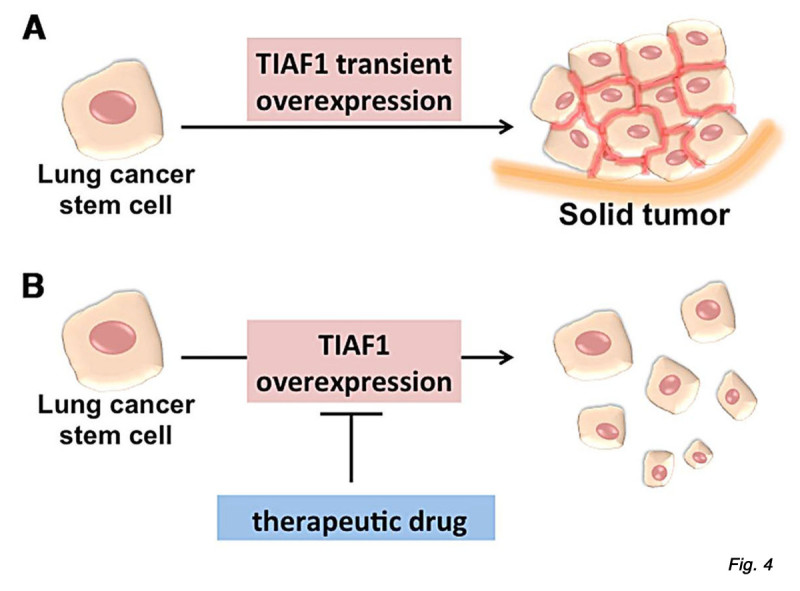
**TIAF1 is a potential target for lung cancer therapy. A.** When cancer cells are transiently overexpressed with TIAF1, a portion of the expressed TIAF1 localizes on the cell membrane/cytoskeleton area or intercellular junctions (marked in red) [37], which probably assists cells to stick to each other that leads to solid tumor formation. TIAF1 is associated with stem cell pluripotency [46]. It is reasonable to postulate that when a lung cancer stem cell starts to grow, it probably needs the expression of TIAF1 to allow cells to stick to each other in order to proliferate. **B**. Therefore, blocking TIAF1 expression by appropriate chemicals, siRNA, or dominant negatives could result in suppression of lung cancer stem cell proliferation.

*TIAF1* gene participates in human embryonic stem cell self-renewal and pluripotency [46], and is also involved in melanogenesis [47] and genome stability [48]. Given its potential role in cancer progression, TIAF1 can be an ideal target for therapeutic drugs. Transiently overexpressed TIAF1 induces aggregation of monocytic cells and other cancer cells *in vitro* [37] (Figure 4). It appears that the overexpressed cell surface TIAF1 serves as a molecular glue, plus integrins and matrix proteins, to allow cancer cells to adhere to each other. Thus, it is reasonable to postulate that blocking TIAF1 expression by appropriate chemicals, siRNA, or dominant negative TIAF1 could result in suppression of lung cancer stem cell growth and progression.

## Authors’ original submitted files for images

Below are the links to the authors’ original submitted files for images. Authors’ original file for figure 1
Authors’ original file for figure 2
Authors’ original file for figure 3
Authors’ original file for figure 4

## Abbreviations

TGF-β: Transforming growth factor beta
TIAF1: TGF-β-induced antiapoptotic factor
WWOX: WW domain-containing oxidoreductase
Smad4: Mothers against decapentaplegic homolog 4
MCT-1: Oncogenic monocarboxylic acid transporter 1
NF-κB: Nuclear factor kappa-light-chain-enhancer of activated B cells
Mdm2: p53 E3 ubiquitin protein ligase homolog
Mdmx: Mdm4 p53 binding protein homolog
JNK1: cJun *N*-terminal kinase
Zfra: Zinc finger-like protein that regulates apoptosis
NSCLC: Non-small-cell lung carcinomas
SCLC: Small-cell lung cancer
CXCR4: Chemokine (C-X-C motif) receptor 4
SDF-1CXCL21: Chemokine stromal derived factor-1/chemokine (C-X-C motif) ligand 12
ECM: Extracellular matrix
Aβ: Amyloid beta
APP: Amyloid precursor protein
siRNA: Small interfering RNA
TREG: Regulatory T cells
TβRII: Type II TGF-β receptor
TNF: Tumor necrosis factor.
