# Supplementary figures and images for: Self-aggregating TIAF1 in lung cancer progression
Source: Transl Respir Med. 2013 Feb 28;1:5. doi: 10.1186/2213-0802-1-5 (PMC6733429; doi:10.1186/2213-0802-1-5)

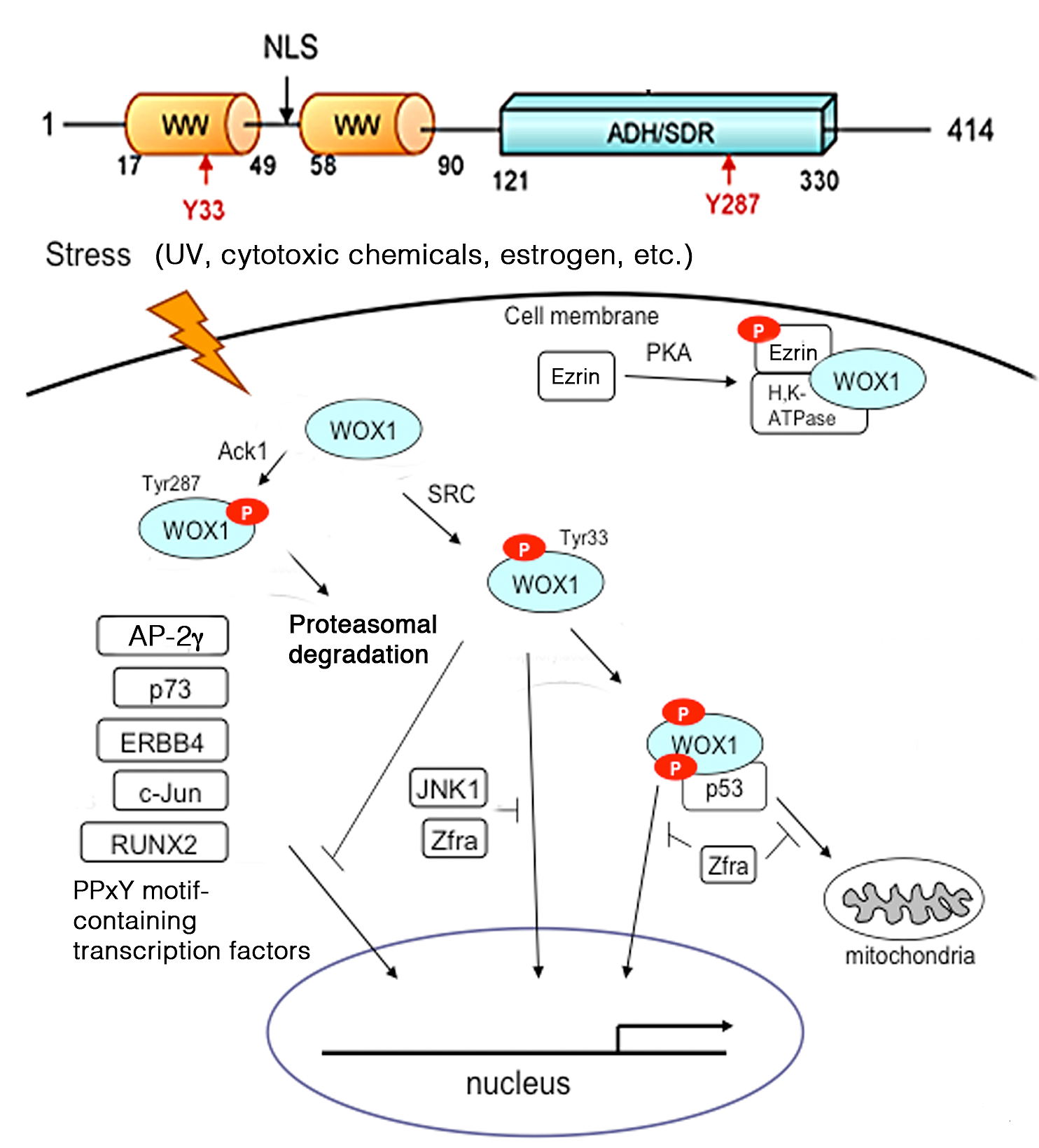

Supplement: Supplementary file 1 — Authors’ original file for figure 1 [file 40247_2012_5_MOESM1_ESM.tiff]

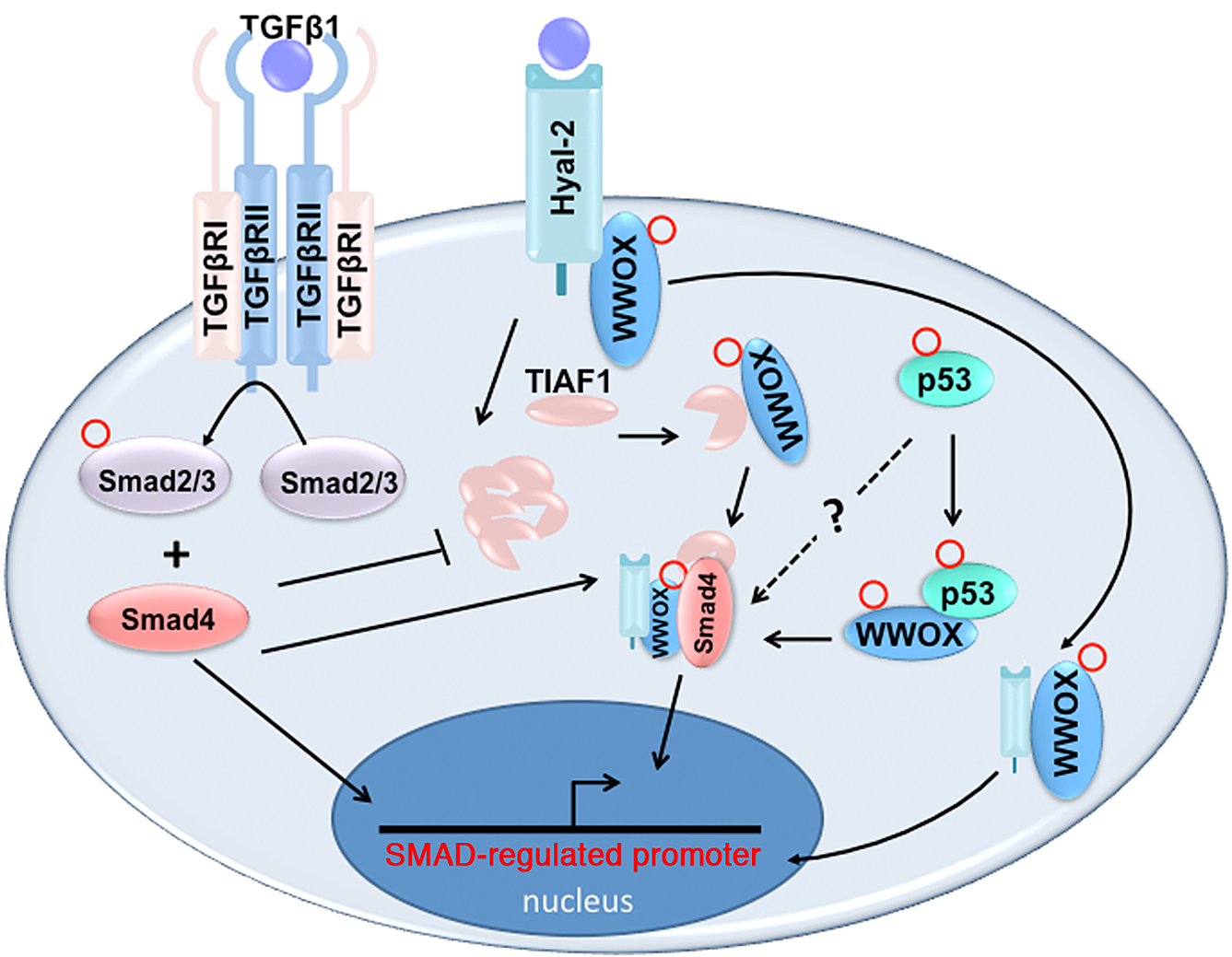

Supplement: Supplementary file 2 — Authors’ original file for figure 2 [file 40247_2012_5_MOESM2_ESM.tiff]

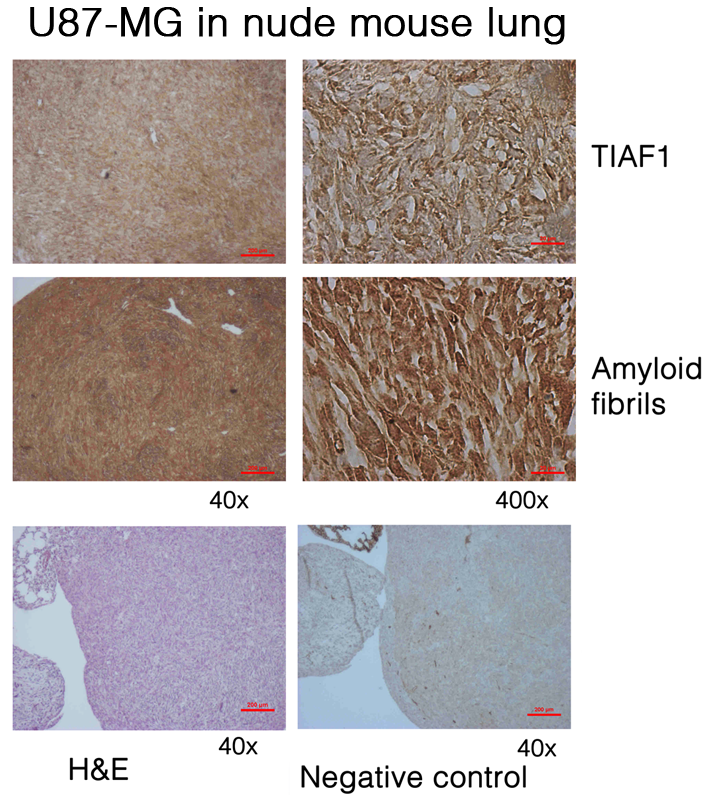

Supplement: Supplementary file 3 — Authors’ original file for figure 3 [file 40247_2012_5_MOESM3_ESM.tiff]

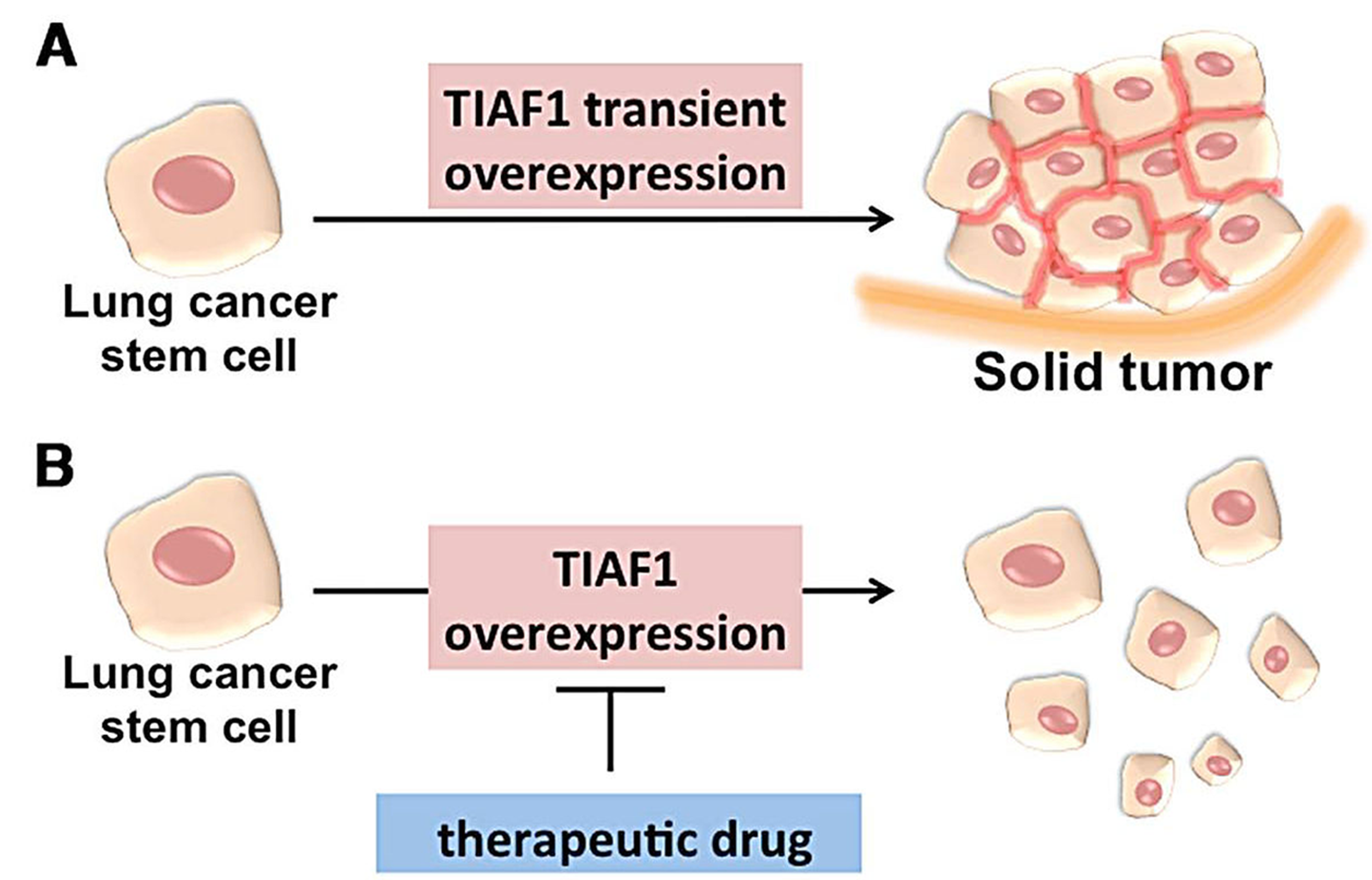

Supplement: Supplementary file 4 — Authors’ original file for figure 4 [file 40247_2012_5_MOESM4_ESM.tiff]
